# Surgery of the Stomach

**Published:** 1904-06-25

**Authors:** 


### Progress in Medicine and Surgery.

### SURGERY OF THE STOMACH.

**Perforating Gastric and Duodenal Ulcer.**—English[1] has analysed the facts connected with fifty consecutive cases at St. George's Hospital, excluding those cases in which moribund patients with perforated ulcers were admitted and in which no operation was performed. As regards the signs of impending perforation he has shown that in a certain proportion of the cases there were a progressive increase of pain and vomiting for a few hours or even for two or three days preceding perforation. In three cases perforation occurred in patients already in the hospital, and all three patients died. The signs of perforation are generally sufficiently clear; the sudden intense and abdominal pain is the most important of these, while vomiting was present in over 75 per cent. of the cases. During the stage of reaction the patient may show few signs, and in one case a man walked into the hospital merely complaining of "stomach-ache," although he had general peritonitis from a perforated duodenal ulcer. English considers that not only morphine, but also stimulants and strychnine may produce such temporary improvement as to mask the symptoms and to lead to the postponement of an urgent operation. The presence or absence of liver dulness is of no value in the diagnosis of perforation, for in eleven out of forty-three cases the liver dulness was normal in spite of perforation having occurred. In the case of gastric ulcers the percentage of recoveries was 52, and in the case of duodenal ulcers it was 25. The proportion of recoveries is increasing, for of the first ten patients only three cases recovered, while of the seven cases admitted during 1903, six have recovered. MacLaren[2] says that rigidity or tension of the abdominal muscles is the key-note to the early recognition of peritoneal perforation from whatever source. The pain may subside, the temperature may not be elevated, but the rigidity continues even up to the end. D'Arcy Power,[3] however, says that rigidity is only marked in the later stages and quotes cases to show that the abdomen may remain soft and may move freely in spite of an acute perforation, but this absence of rigidity seems to be more common in acute duodenal than in acute gastric perforation. Other cases are reported by Lucas,[4] Smeeton,[5] and Tonkin.[6] Maunsell[7] calls attention to the following points: (1) Men and women of all ages are liable to perforation of a gastric ulcer. (2) The history may, but very often does not, disclose previous ulceration. (3) Shock is a variable sign and tends to pass off to a great extent within a few hours. Twelve to 24 hours after perforation the patient feels much better, but examination reveals a rigid abdomen and lack of proper abdominal respiratory movements. (4) The temperature in the vast majority of cases is either normal or subnormal during the first 24 hours. (5) The pulse-rate in some women is frequent, in others infrequent, in the majority of men it is infrequent, ranging between 60 and 90, unless the case has been left so long as to practically ensure failure from any form of treatment. (6) The character of the pulse is not hard and "wiry," except in some long-neglected cases. (7) It is in most cases impossible to clearly demonstrate fluid in the flanks by percussion. (8) The "stomach-note" is still present, or is even exaggerated, since perforation leads to paralytic distension of the stomach with gas, not to collapse of that viscus. (9) The absence or presence of liver dulness is too uncertain a guide to be of much assistance. (10) The tongue is always moist and white, or slightly yellow, not brown, dry, and cracked, until after the lapse of many hours. This is of very great importance. If there is a reasonable probability that perforation has taken place the abdomen should be opened as soon as arrangements can be made.

**Chronic Gastric Ulcer.**—Moullin[8] says the ideal operation for gastric ulcer is undoubtedly excision; but it can rarely be performed, either because the ulcer or the scar is too near to the pylorus or because the ulcer has attained such a size, from having been left too long, that such a proceeding would interfere with the shape of the stomach. Of 23 cases, gastro-jejunostomy was performed in 20, pyloroplasty in two, and excision of the ulcer with gastro-jejunostomy in one. The operation adopted in nearly all cases was a posterior retro-colic gastro-jejunostomy with, in addition, a lateral anastomosis, between the adjacent sides of the duodenal loop. The lateral anastomosis enables the patient to be fed at once by introducing food directly into the bowel · it effectually prevents the possibility of such a thing as a vicious circle, and it renders the portion of the bowel which descends from the stomach through the opening in the mesocolon sufficiently rigid to check the rest of the small intestine from passing through the opening into the lesser peritoneal cavity. In every case the patients were given immediately before the operation one-thirtieth of a grain of strychnine with either one-hundredth of a grain of digitalin or two grains of sodio-salicylate of caffeine hypodermically, and an enema of eighteen ounces of black coffee with two ounces of brandy. Maunsell[9] points out that it is not always rational to advise excision of the ulcer for the following reasons :—(1) It is not usually easy to locate the position of an ulcer at operation. (2) Ulcers are most commonly situated upon the posterior wall, and are hard to reach, more especially if the base is adherent to the pancreas, etc. (3) Ulcers are very frequently multiple. (4) Ulcers are very frequent in the pyloric antrum and at the pylorus, where excision would lead to narrowing unless combined with pyloroplasty, which is not always advisable or readily performed. (5) The cause of the ulceration is in no way mitigated by the procedure, and the trouble may recur at a future date. There are at least two great objections to pyloroplasty :—(1) It has been found that where active ulceration is present at the pylorus, contraction frequently follows upon a pyloroplasty, and further operation becomes necessary. (2) It is very difficult, clinically, to differentiate gastric and duodenal ulceration, or perhaps both lesions may be present, and pyloroplasty could have no beneficial effect upon the duodenal lesion. Gastro-enterostomy has been found at once the most useful and the safest operation when all things are considered—it has a mortality of less than 5 per cent. With reference to hæmatemesis or melæna in cases of gastric ulceration he suggests the following :—(1) In all cases in which there is no history of previous ulceration, although the amount of blood lost may be a pint or more, it appears wiser not to operate. (2) If the bleeding should recur in some hours, and still be copious, the patient showing signs of continued loss of blood, operation should be considered. (3) If there is a history of previous ulceration, which has not been thoroughly treated, the advice would be the same ; but if the history reveal previous failure of non-operative treatment, operation should be very seriously considered. 4. If the history reveal recurring attacks of severe hæmorrhage, operation should be considered imperative. 5. If in a first attack of acute hæmorrhage, or in recurring hæmorrhage of even a much smaller amount, structural changes in the stomach, such as pyloric stenosis, are clinically evident, operation appears to be the only sensible line of treatment. 6. In those cases where it is not deemed necessary to operate, we may advise 10 to 30 drops of adrenalin chloride solution by mouth, to be repeated in four hours.

**Perigastric Adhesions** are often preceded by symptoms characteristic of gastric ulcer or gall-stone colic. Paton[10] says that the chronic trouble which is left after these acute symptoms have passed off is not easily amenable to ordinary treatment. Of this residual trouble pain is the most common and characteristic symptom. It may be constant or intermittent, but one of its most marked features is the way in which this pain is not infrequently almost confined to one spot. It may not be influenced by food, but exercise and position may affect it. Local tenderness over a limited area is very frequent, and areas of dulness are exceptionally found. When such adhesions are suspected, an exploratory incision should be urged. Mayo Robson[11] says this operation of gastrolysis can be performed with very small risk—the adhesions must be broken down, for as the trouble is mechanical it is quite clear that general treatment can do no permanent good.

**Cancer of the Pylorus.**—Mayo[12] says 70 per cent. of all gastric carcinomata involve the pyloric portion. The lymphatic supply is important from the operator's point of view. In a general way the lymph channels follow the blood-vessels. On the lesser curvature the blood- and lymph-vessels lie in the wall of the stomach itself, and, as pointed out by Mikulicz, it is necessary in every case of pyloric cancer to remove all the lesser curvature to the gastric artery. For convenience, this situation on the lesser curvature for the beginning of the line of excision may be called the Mikulicz point of election. Cuneo showed that there are but few lymph glands along the greater curvature, and these are confined to the pyloric region. These glands, with the blood-vessels, are set at some distance from the greater curvature, thus enabling rapid expansion and contraction of the stomach, without interference with the circulation. The lymph stream in this situation flows from left to right, and does not drain more than one-third of the adjacent stomach, two-thirds going into the lymph channels of the lesser curvature. In the immediate vicinity of the pylorus, however, it drains its fair share. The lymphatics of the greater and lesser curvatures enter the deep receiving glands about the cœliac axis on the anterior surface of the aorta. Cuneo practically demonstrated that the fundus and two-thirds of the greater curvature are free from lymphatic involvement in cancer of the pylorus.

[1] Lancet, Dec. 19, 1903, pp. 1707 and 1737. [2] Med. Rec., Jan. 16, 1904, p. 117. [3] Clin. Jour., Dec. 2, 1903, p. 98. [4] Bir. Med. Rev., Nov. 1903, p. 697. [5] Brit. Med. Jour., Dec. 19, 1903, p. 1588. [6] Lancet, Jan. 9, 1904, p. 91. [7] Dub. Jour. of Med. Sci., Jan. 1, 1904, p. 1. [8] Lancet, Jan. 9, 1904, p. 95. [9] Dub. Jour. of Med. Sci., Jan. 1, 1904, p. 8. [10] Lancet, Feb. 6, 1904, p. 358. [11] Clin. Jour., March 2, 1904, p. 319. [12] Ann. of Surg., March, 1904, p. 321.

